# Rational Optimism

**DOI:** 10.1007/s11406-024-00758-w

**Published:** 2024-07-25

**Authors:** Matthew F. Wilson, Tyler J. VanderWeele

**Affiliations:** 1 Philosophy, John Brown University, 2000 West University Street, Siloam Springs, AR 72761 USA; 2 Human Flourishing Program, Harvard University, Cambridge, MA 02138 USA; 3 John L. Loeb and Frances Lehman Loeb Professor of Epidemiology, Harvard T.H. Chan School of Public Health, Departments of Epidemiology and Biostatistics , 677 Huntington Avenue, Boston, MA 02115 USA

**Keywords:** Optimism, Unrealistic optimism, Comparative optimism, Dispositional optimism, Epistemic rationality

## Abstract

Optimistic beliefs have been criticized by philosophers as being irrational or epistemically deficient. This paper argues for the possibility of a rational optimism. We propose a novel four-fold taxonomy of optimistic beliefs and argue that people may hold optimistic beliefs rationally for at least two of the four types (resourced optimism and agentive optimism). These forms of rational optimism are grounded in facts about one’s resources and agency and may be epistemically justified under certain conditions. We argue that the fourth type of optimism in our taxonomy (perspectival optimism) is not subject to epistemic scrutiny in the same way. It is better evaluated on practical and moral grounds. This paper advances the discussion of optimism within both the philosophical and psychological literatures by providing a compelling and philosophically rich taxonomy of optimism that clarifies the sometimes-competing forms of optimism identified by psychologists. This advances the field by putting forward cases of epistemically justified optimism, in contrast with unrealistic optimism, that is sometimes justified for its instrumental or adaptive characteristics, and also by highlighting a form of optimism, perspectival optimism, that is not being considered in the mainstream optimism literature in psychology. The paper concludes by suggesting several avenues for future empirical and philosophical research.

## Introduction

Philosophers often view optimism with considerable suspicion. Optimism is sometimes taken to imply a tendency to think that things will turn out better than what the objective facts warrant. As such, optimism is often viewed as an epistemic deficiency. Philosophers have thus devoted more reflection and analysis to the subject of hope^1^, or, alternatively, to pessimism^2^. Optimism has been neglected in philosophy, as it characteristically will involve a wrong way of understanding reality and usually some form of irrationality. Or so it is thought. In English, the word “optimism” is used in a variety of ways with different nuances of meaning. This paper advances the research on optimism by delineating the conceptual boundaries on four distinct types or forms of optimism that humans may possess. We argue that while some of these forms do entail epistemic deficiency, others do not and may be epistemically warranted. We argue, in other words, that there are in fact types of rational optimism. Furthermore, we argue that one type of optimism (which we call perspectival optimism) does not consist in beliefs or predictions about the future and is thus not open to epistemic scrutiny in the traditional sense. Researchers, including philosophers, have often overlooked this form of optimism, and instead have focused on optimism as an erroneous or unwarranted belief. Finally, although the instrumental value of optimism for human adaptation and well-being has been well documented, few have considered its possible importance and connections to morality, which we do here.

The paper is structured as follows. We begin by briefly motivating the issue of optimism’s epistemic rationality (or irrationality) from considerations that arise in the social-psychology literature on optimism. We then distinguish four types of optimism based on the relevant grounds or perspectives that underly a person’s positive expectations or positive views towards the present or future. We call these types *groundless optimism*, *resourced optimism*, *agentive optimism*, and *perspectival optimism*. We believe this four-fold taxonomy is novel to both psychology and philosophy. We then consider the circumstances under which these forms of optimism might be considered rational or irrational, and we also argue that the rational forms of optimism may be worth cultivating on moral and practical grounds. While the focus of this paper is on optimism, not on hope, we do offer some comments on the distinctions between hope and each of the four types of optimism that we describe, and especially with what we call agentive and perspectival optimism, which bear some similarity with, but are nevertheless distinct from, hope. We conclude the paper by offering suggestions for how these distinctions may inform both the empirical study of optimism within psychology and biology, as well as future philosophical reflection on of the role of optimism in human life.

## Optimism in Psychology

Let us begin our investigation of optimism through the door of psychology. Although philosophers have given some attention to optimism, psychologists have taken a much greater interest.^3^ This is in part because optimism has been shown to be widespread and have practical benefits that are empirically demonstrable, including but not limited to improved mental and physical health.^4^ Philosophers, on the other hand, have treated optimism more skeptically, tending to focus on its epistemic weaknesses. As Eagleton aptly states, “There may be good reasons for believing that a situation will turn out well, but to expect that it will do so because you are an optimist is not one of them. It is just as irrational as believing that all will be well because…it has just rained for three days in a row.”^5^

Both psychologists and philosophers, however, do acknowledge that optimism can be epistemically deficient, as optimistic expectations are commonly unsupported by the available evidence, or held in the face of counterevidence. When we say that optimism may be “epistemically deficient” or “epistemically irrational” we are referring to a common distinction between epistemic rationality and instrumental rationality made in philosophy. We follow Thomas Kelly (2003, p. 612) in defining epistemic rationality as “roughly, the kind of rationality which one displays when one believes propositions that are strongly supported by one’s evidence and refrains from believing propositions that are improbable given one’s evidence.” Our inquiry into the epistemic rationality of optimism is therefore not to be confused with instrumental rationality, which is the rationality one displays in pursing means to one’s ends.^6^ Thus, if it turns out that holding optimistic beliefs makes people healthier, happier, more successful, etc., this does not mean that holding those beliefs is epistemically rational, even if it may be instrumentally rational to do so.^7^ As Jefferson et al. note, “Epistemically irrational beliefs and predictions can be either true or false, but what makes them irrational is that they were not formed on the basis of (sufficiently robust) evidence or are insufficiently responsive to evidence after being adopted” (2017, p. 7).

In the literature on optimism there is no consensus on what optimism is, but the majority of psychologists and most philosophers tend to treat optimism as a belief state: one of expectancy. Optimism is typically defined as an expectancy that future events or states of affairs will turn out positive (Avvenuti et al., 2016; Carver et al., 2010; Kadlac, 2015; Pettit, 2004; Sharot, 2011a; Weinstein, 1980). Of course, not all agree on this definition. Day (1969) conceived of optimism as a disposition to hope, but in a rather extreme form.^8^ Roberts (2007) holds a view that optimism is a type of mood. But for now, let us take the most common definition as a starting point: namely, that optimism is a belief state constituted by expectations of positive outcomes or states of affairs in the future. The word optimism can of course also refer to a trait – optimism in its dispositional form.^9^ An optimistic *person* is thus one who is disposed to form optimistic beliefs.

Setting these definitional issues aside, let us proceed by examining some of the epistemically questionable forms of optimism. Psychologists have long studied the human tendency to hold unjustified positive future expectations, and this commonly became to be called “unrealistic optimism” (Weinstein, 1980, 1989).^10^ From the perspective of many, unrealistic optimism is a kind of *positive illusion* (Taylor, 1989; Taylor & Brown, 1988). It is a “positive” mental state because it is associated with a host of practical benefits, but it is also acknowledged to be illusory from an epistemic standpoint.

Shepperd et al. distinguished two types of “unrealistic” optimism (Shepperd et al., 2013).^11^ The two forms of unrealistic optimism they identify are absolute and comparative unrealistic optimism.^12^*Absolute unrealistic optimism* consists in having expectations about positive future events or states of affairs that are unrealistic as compared to their *objective likelihood*. A gambler, for instance, may hold unrealistic expectations about his chances of winning a game when compared to the game’s actual odds.^13^*Comparative unrealistic optimism*, by contrast, occurs when people make unrealistic predictions about positive future events occurring for themselves *relative to others*.^14^ For example, a person may believe she has a better chance of not getting cancer than other people (one commonly sees this in smokers), or that her chance of getting divorced is less than that of others. Such comparative optimism is unrealistic when the assessment compared to other people is in fact incorrect. Psychologists note the difficulty of proving that any particular individual is wrong in holding comparatively optimistic beliefs, because individual differences in each person’s life can at times make such beliefs warranted.^15^ But it is also easy to prove the existence of comparative unrealistic optimism within populations. As Jefferson et al. note, “when 70% of the population take themselves to be less likely to be divorced than the average person, they cannot all be correct” (2017, p. 6).^16^ Likewise 94% of college professors cannot all perform above-average work, like they say they do (Cross, 1997). At least some of those people must be unrealistically optimistic, regardless of individual differences.

Optimistic beliefs can be thus unrealistic in at least two ways: either absolutely or comparatively.^17^ In either sense, they are epistemically deficient. So what is realistic optimism? The authors cited above do not seem to provide an adequate definition of realistic optimism, and, as noted above, philosophers tend to view optimism as involving a form of irrationality. In Jefferson et al.’s discussion, they at one point attempt to defend the notion of realistic optimism by pointing to the fact that *dispositional* optimism is a generalized tendency to expect positive outcomes and is therefore not dependent on one’s expectation for any specific outcome.^18^ In other words, they point to the fact that human dispositions are not subject to epistemic scrutiny per se. To evaluate a disposition as rational or irrational would be to make a category mistake. But dispositional optimism still disposes one to generate optimistic beliefs, so this defense seems to miss the point.

The idea of realistic or rational optimism may at first appear paradoxical. There is, however, a body of empirical research that shows people of higher education, and higher socio-economic status, are, on average, more optimistic than others (Heinonen et al., 2006). Why is this? The obvious reason seems to be that they have *grounds* to be more optimistic, given that they come from a position of privileged resources. In other words, if we get away from the term “realistic”,^19^ we can think instead in terms of whether a person’s optimism is warranted or *grounded* – i.e. whether one has good reasons for one’s optimism. Whereas the literature focuses almost exclusively on the variety of ways that optimism can be unwarranted, the conceptual analysis of optimism offered below provides insight into the ways that optimism can in fact be grounded in facts sufficient to justify those beliefs as rational. We also argue that one form of optimism exists that is not subject to epistemic appraisal in the way we normally think of it.

## Four Forms of Optimism

The analysis that follows conceptually divides optimism into four types or forms. The first two types of optimism are grounded in reasons, and the expectations formed on the basis of those reasons therefore may or may not be epistemically justified (these we will call “resourced” and “agentive” optimism). The next and third type of optimism is groundless (which we accordingly refer to as “groundless” optimism) and can never be epistemically justified. The fourth and final form of optimism is neither grounded nor ungrounded in reasons, because it consists in a way of construing or seeing the world. It does not necessarily concern a judgement or expectation about the future (we refer to this as “perspectival” optimism and discuss it last). For now, let us focus on the first three forms of optimism that do involve expectations about the future.

The first form of grounded optimism we call *resourced optimism*, in which a person holds positive expectations about future events that are grounded in his or her own resources (such as education, experience, financial assets, etc.). We might, for example, imagine a well-educated entrepreneur who has already been successful with several different start-ups. If she were to form an optimistic belief about the prospects of her newest start-up, and this positive expectation were grounded in her previous experience, lessons learned, financial resources, etc., then it seems such a belief might qualify as both optimistic and epistemically warranted, or rational, insofar as the expectation is grounded in good reasons.

The second form of grounded optimism we call *agentive optimism*, in which a person holds positive expectations about a future event because at least some of that good future depends on his or her actions, and he or she is committed to exerting considerable effort towards bringing about the positive outcome. Such optimism is grounded not so much in an agent’s resources as in her commitment and determination to expend significant effort – her agency – in pursuing the desired positive future outcome. This type of optimism is grounded in a person’s strong determination to see a positive state of affairs come about.^20^ This second type of grounded optimism is conceptually distinct because it is possible to possess regardless of the resources that one starts with.

The distinction that Shepperd et al. make between absolute and comparative optimism could apply to both categories as well. For example, it is conceivable that an entrepreneur could conceive of her expected success in comparative terms. In other words, she could be optimistic that she is less likely to fail compared to most entrepreneurs because of her resources, etc., instead of just expecting to succeed absolutely because of those resources.

Moving on from these forms of grounded optimism, let us consider a third form of optimism – what we call *groundless optimism*. Whereas the first two forms are grounded in a person’s resources or personal agency, this third form of optimism is a positive expectation about the future that is held without epistemic grounds. In the example we have been using, it is possible to conceive of an entrepreneur who does not have experience or financial resources and who is not particularly committed to working hard. If she nevertheless simply expects to succeed, her optimism may be groundless. Groundless optimism is never epistemically justified.

Each of these first three form of optimism – resourced, agentive, and groundless – can present themselves either as a specific belief state or as a disposition to form such beliefs, perhaps arising from one’s actual resources, or one’s sense of agency, or one’s temperament, or some combination of these and other factors. In other words, each can be conceived of in an episodic or dispositional form.

The fourth and final form of optimism, however, is not a belief state. Understanding it requires us to make a conceptual shift because it does not consist in holding a belief or forming an expectation about the future at all. It rather is a particular way of construing or “seeing” the world.

Consider that resourced, agentive, and groundless optimism might all be classed into a genus called *expectancy optimism*, because each has an *expectation* about the future at its core. This is the genus of optimism that is discussed almost exclusively in the literature.^21^ However, we argue that there is another type of optimism frequently found in persons that does not always concern future expectations. In fact, English dictionaries define optimism in such a way as to suggest that there is more to optimism than just expectations about the future. Consider the following two definitions of optimism:

**Optimism** the tendency to be hopeful and *to emphasize or think of the good part in a situation rather than the bad part*, or the feeling that in the future good things are more likely to happen than bad things (Cambridge English Dictionary, emphasis added)^22^

**Optimism** an inclination *to put the most favorable construction upon actions and events* or to anticipate the best possible outcome (Merriam-Webster Dictionary, emphasis added)^23^

The italicized parts of these definitions are ways of emphasizing, construing or “seeing” the world. Taking these definitional clues as a starting point, we suggest that this fourth form of optimism consists in taking a certain perspective, or of making salient certain features of a situation, rather than in forming belief expectations about the future. Consider the following statements:

“The glass is half full” (vs. half empty).

My failure to get the job provided me a good learning opportunity.

I have a 20% chance of beating this disease. That’s better than 5%!

Each of the foregoing statements expresses optimism. Each exemplifies a positive perspective or a positive way of construing a situation and what it is about. Each statement could also be reconfigured to display a pessimistic perspective on that same state of affairs. Yet none of these statements makes a prediction or necessarily expresses an *expectation* about the future.

We therefore define *perspectival optimism* as a state where a person gives attentional focus to the positive or good aspects of any intentional object. That object may be some future event, but it need not be. Thus, perspectival optimism is not a belief state; it is not a probability judgement; and it does not necessarily concern the future. It is rather a *description* of an agent’s mental activity – how, and on what, he or she focuses attention. It describes the way an agent “sees” a situation as she focuses her attention on its positive or good aspects. Perspectival optimism is therefore not subject to the same sort of epistemic scrutiny as the other three forms of expectancy optimism – although, we will show, it is not entirely unrelated to broader epistemic concerns.

Perspectival optimism can also be considered in both its occurrent and dispositional forms. The occurrent form might involve a person deliberately focusing his or her attention on the positive aspects of a situation, or it could happen more spontaneously. By contrast, someone possessing the disposition would *characteristically* focus on or make salient the positive aspects of the intentional objects that she thinks about. Regardless of how the perspective comes about, the end result is that an agent’s attention is focused on what is good in the context under consideration.

Perspectival optimism need not concern the future, but at times it may concern the future. We can imagine someone saying, “When thinking about the future, I always concentrate on what is likely to turn out positive.” Such future-oriented perspectival optimism bears some similarity to the other types of expectancy optimism discussed above, a point to which we will return below, but it remains distinct as a type and genus of optimism.

## Rational Optimism

We have now established a taxonomy of optimism with two genera: expectancy optimism and perspectival optimism. Expectancy optimism has three types, each of which are constituted in part by positive future expectations. These types are resourced optimism, agentive optimism, and groundless optimism. Perspectival optimism, by contrast, concerns the way that an agent focuses her attention on positive or good aspects of situations or circumstances; it does not concern expectations. We now consider whether and how each of these forms of optimism might be epistemically warranted and thus considered to be epistemically *rational*. We analyze the conditions under which they might be judged to be so.

Groundless optimism, wherein one holds positive expectations about future events that are without grounds, cannot be said to be rational. If asked, “Why do you expect that your chosen horse will win the race?” and one answered, “I don’t know; I just feel lucky”, then the optimism is groundless. Such cases of groundless optimism are rightly criticized as epistemically deficient, regardless of their instrumental value.

However, it is important to distinguish groundless optimism from what might be called *unspecified* or *generic optimism*, wherein grounds for positive expectations are not stated or offered but could be offered upon questioning. For example, a person might optimistically say, “On the whole, I expect my kids to turn out well.” No grounds may be offered, but the expectation may not in fact be groundless. It may just be an incomplete or under-developed thought. When the person makes the utterance and is questioned, she may be able to provide reasons like financial resources, parenting skills, or intended effort, thereby constituting resourced or agentive optimism. In determining, therefore, whether an unspecified statement of generic optimism is in fact groundless optimism, or whether epistemic grounds may be present, further questioning may be needed.

Next, recall that the two types of grounded optimism are resourced optimism and agentive optimism. Each is grounded in facts or beliefs about one’s resources or in one’s agency. But the fact that an agent has grounds for a positive expectation does not necessarily mean that the belief is warranted or rational. It is thus necessary that the beliefs concerning the grounds for one’s optimism be true, and it is furthermore important that the grounds themselves are relevantly proportional to warrant the optimistic expectation. We will refer to these two conditions as the truth test and the proportionality test – tests that must be passed for one’s optimism to be epistemically rational. To illustrate this, it will be helpful to look at some examples.

Imagine a student who says, “I expect to get an ‘A’ on the final exam simply because I am smarter than most students.” This would be an example of resourced optimism. But it might not be rational for two reasons. First, if the person speaking was not in fact smarter than most students, then her beliefs concerning the grounds for her optimism would be false. Her expectation would fail the truth test. Second, we might question whether being smarter *than most students* is relevantly proportional to getting an ‘A’ on a final exam. If the professor grades on a curve, her relative ability compared to most students might be sufficient to warrant her expectations. But if the professor was known to be especially hard and did not grade on a curve, or if good exam performance were to require considerable effort at memorization and the student was unwilling to exert such effort, then being “smarter than most students” might not be relevant to getting an A on the final exam.

As another example, imagine someone saying, “I expect to be able to reach the peak of Mt. Everest without supplies because I am physically fit” (resourced optimism), or alternatively, “I expect to be able to reach the peak of Mt. Everest without supplies because I am going to put in a lot of effort” (agentive optimism). In both cases, the expectation is clearly unreasonable. Why? Both examples exhibit a lack of proportionality between the grounds and the positive expectation. In both cases the beliefs underlying the grounds for optimism may be true (i.e., “I am physically fit” or “I am going to put in a lot of effort”), and one’s fitness and effort certainly are relevant to summiting Mt. Everest. But these grounds do not justify the expectation because they are not proportional to the difficulty of accomplishing such a feat without supplies. Thus, the person’s optimism in this case would not be rational.^24^

Again, for grounded or agentive optimism to be rational, it must be the case that the beliefs underlying the grounds offered are true and relevantly proportional to justify the positive expectations. A yet stronger form of rational optimism might require that not only that the beliefs underlying the grounds offered be true, but also that the beliefs themselves be justified or known to be true. It is beyond the scope of the present paper to offer a full account of epistemic rationality and what justifies beliefs or when beliefs can be considered knowledge, but we would propose that, for any adequate account of epistemic rationality, a possibly overly strong sufficient condition for optimism to be epistemically rational could be that the grounds offered must be known to be true and that they are indeed sufficient to justify the positive expectations. However, since both resourced and agentive optimistic beliefs concern the future with its inherent uncertainty, we do not think it always necessary for one to know that one’s grounds are sufficient to justify one’s positive expectations.^25^ In most cases such knowledge is not possible.

Regardless of one’s judgement concerning these stronger and weaker conditions, it does seem to be possible to have positive expectations about the future that are both grounded and warranted – i.e. rational forms of optimism. Both resourced optimism and agentive optimism may be considered rational under the right conditions.

## Scope of Optimism and Dispositional Optimism

Another important consideration in determining the rationality of optimism is its scope. Scope is simply the range or set of activities, events, or outcomes that a particular optimistic expectation ranges over. Scope can be either narrow or wide. For example, consider the expectations people form about a particular outcome occurring, an event happening, or a project succeeding. The range of these expectations is narrow, and thus so is the scope.

By contrast, wider-scoped optimism often ranges over entire domains of life or sets of events considered together. For example, someone might be optimistic about his marriage – that the relationship will be sustained for life and that it will be full of positive experiences and a deep sense of commitment and emotional connection. Importantly, someone may believe this without necessarily thinking that every encounter with his wife will be positive. But considered as a domain of life that will contain many different types of events and situations, one can be optimistic that the states of affairs and events that make up one’s marriage will, on the whole, be positive. The same could be true of one’s relationships more generally, or of one’s career, or of other domains of life. Of course, such beliefs could again be grounded or groundless.

The broadest forms of expectancy optimism could range over the whole of one’s life, or even more generally, the future of the entire world. Eagleton (2015), for example, identifies the progressivist movement of Spencer and Comte as embodying a global type of optimism – they were optimists about world Progress (with a capital P). Eagleton is notably critical about this kind of optimism on epistemic grounds.

The epistemic rationality of wide-scoped optimism is often difficult to assess because it is usually hard to know whether the reasons for such optimism can be warranted. There are two considerations that make such judgements difficult. First, with wide-scoped optimism, it may be difficult to specify what exactly the positive expectations are. What is it, precisely, that one expects in life when one is expecting one’s marriage to be good? Is it merely having mostly positive interactions with one’s wife, or does this extend to good family relations with one’s in-laws as well? Moreover, what exactly might be understood by “mostly positive interactions”? To what *extent* are positive interactions expected to exceed the negative interactions? Wide-scoped positive expectations can only be grounded and justified if the extent of these positive expectations are at least somewhat clear.

Second, even if the set of expectations is clear, it is often very difficult to establish whether the grounds given for a wide-scoped optimistic belief can be sufficiently relevant and proportional to justify it. It generally seems that the wider the scope, the more difficult it is for one’s expectations to be adequately justified. However, with a clearly specified scope, and extent, of one’s positive expectations, one’s resources and agency might, in some circumstances at least, be viewed as justifying a relatively wide-scoped optimism. One way, then, to make wide-scoped optimism more rational is to work towards specifying more precisely what one’s positive expectations are, and then proceeding to evaluate the grounds for those optimistic beliefs.

The width or narrowness of a person’s optimistic beliefs should not be confused with the disposition to form optimistic expectations (in the case of expectancy optimism) or a disposition to focus on the positive (in the case of perspectival optimism). Just because a person has a very wide-scoped optimistic belief *occurently* – say that his whole life will turn out well – does not necessarily make him a dispositional optimist. Such a person may expect positive things for the totality of his life today because of experiencing a beautiful sunrise, but on most other days his expectations might be neutral or even pessimistic. Dispositional optimism requires that a person have the tendency or habit to form optimistic beliefs with regular frequency. Such people are *characteristically* optimistic. Unfortunately, the scope of a person’s optimism and the disposition to be optimistic are issues that can be easily conflated.

Psychologists have shown considerable interest in studying the dispositional aspect of optimism, and there is empirical evidence that some people manifest such dispositions (Scheier & Carver, 1987, 1992). To our knowledge, however, psychologists have not considered scope as a dimension of dispositional optimism. In the most popular measurement instrument, the two aspects are blended together. Dispositional optimism is thus commonly assumed to be optimism that is both characteristic of an agent *and* has a wide scope. It is popularly measured through the “Life Orientation Test – Revised,” or LOT-R (Scheier et al., 1994), which is the most widely used psychological measure of optimism.

We wish to avoid confusion over the distinctions between occurrent and dispositional optimism, on the one hand, and wide and narrow scope optimism, on the other. We therefore refer to the optimism measured in the LOT-R as *generalized optimism*. We define generalized optimism as a kind of optimism that applies to one’s life as a whole (wide-scoped) and is a disposition to make such optimistic assessments.

The items in the LOT-R that attempt to capture generalized optimism, assessed by self-report, are as follows:

Overall, I expect more good things to happen to me than bad.

I’m always optimistic about my future.

In uncertain times, I usually expect the best.

If something can go wrong for me, it will. (reverse coded)

I hardly ever expect things to go my way. (reverse coded)

I rarely count on good things happening to me. (reverse coded)

The optimism reflected in the LOT-R statements is not only generalized in the sense of applying to the whole of life with no delineation of domain, but, in the classification described above, it is also *unspecified* or generic in terms of its grounds. The assessment does not provide insight into whether people have good reasons, or any reasons, for their optimism. Thus, the various forms of optimism discussed above – groundless optimism, resourced optimism, agentive optimism – could each contribute to a sense of agreement with the generic LOT-R optimism statements. Even certain forms of perspectival optimism could arguably contribute. With perspectival optimism, when the object is in the future, one might focus one’s attention on those aspects of the future that are indeed likely to be good. This focus could, in turn, give rise to one’s general positive expectations about the future. Thus, when we speak of someone of possessing generalized optimism, or being an optimistic person, this may arise from some combination of groundless, resourced, agentive, and perspectival forms of optimism.

Let us now consider whether such a generalized optimism may be rational. There are arguably two issues at stake. First, there is a question of whether a *wide-scoped* optimism is ever rationally justified given the difficulties and uncertainties of human life. Second, there is a question of whether the *disposition* to hold a wide-scoped optimism can be epistemically justified.^26^

To answer the first question, we can imagine two possible but extreme forms of generalized optimism. One the one hand, we can imagine a generalized optimism that arises purely from groundless optimism, which is obviously irrational. On the other hand, we can imagine a generalized optimism arising *purely* from resourced and agentive optimism. However, even this second scenario leaves open the question of whether a person’s resources and agency can rationally justify being a generalized optimist, especially given our proportionality test. It seems possible that a person with substantial financial, physical, social, and character-based resources may be epistemically justified in holding the expectancies of a generalized optimist, at least as expressed in statements like those found in the LOT-R, but assessing this would require further inquiry into the scope and extent of his or her positive expectations and also his or her recognition of the intrinsic uncertainties of life. As another example, a person who held a certain theistic worldview may be optimistic that God will ultimately work things out for the good thereby providing religious grounds that might epistemically justify a generalized optimism, at least from within the standpoint of that worldview, though others might dispute whether their beliefs were in fact true.^27^

As noted above, holding a wide-scoped form of optimism does not require the expectation that nothing at all will be negative in one’s life. People who are well-resourced, or religious individuals, may hold a kind of generalized optimism even while fully recognizing that not every event in the future will turn out positive. The expectancies of generalized optimism may be held in aggregate or from an all-things-considered perspective. However, for such optimism to be rational, it must at least be somewhat clear to the optimistic person what the generalized positive expectations are and what the grounds are for holding them.

As for the *disposition* to form wide-scoped optimistic beliefs, it was noted above that dispositions as such cannot themselves be epistemically rational or irrational. Again, that would be a category mistake. However, such dispositions may be grounded in irrational beliefs, or may give rise to irrational beliefs, making them epistemically relevant for our lives. Thus, when a wide-scoped optimistic disposition is operative, we can still question whether the positive expectations in each instance are rationally grounded or not, and we can thus also comment on dispositions towards irrational beliefs and expectations as being epistemically problematic.

We suspect that most generalized optimists manifest at different times both grounded and groundless forms of optimism. We also suspect that, in most cases, generalized optimism arises in part from perspectival optimism. It also seems plausible that a habit of seeing the positive within any situation can lead to one forming wide-scoped expectations for one’s life more generally.

## Practical and Moral Considerations

In the previous section we discussed two criteria that are relevant to whether *expectancy* optimism may be rational. Let us now consider the rationality of perspectival optimism. Perspectival optimism does not principally concern an expectation about the future (a belief state). It is rather a matter of attentional focus. It would thus seem less susceptible to epistemic scrutiny than expectancy optimism. There are, however, cases in which epistemic assessment is arguably appropriate even for perspectival optimism. Although it is principally a matter of attentional focus, if one focuses on seemingly positive aspects of a situation that are known or believed to be false, then this too would be an epistemic deficiency. Likewise, certain extreme cases of perspectival optimism might be judged irrational both on both epistemic and practical grounds. One might envision an extreme Pollyannaish form of perspectival optimism in which the whole of one’s attentional focus is only on whatever good one might find in every situation, always ignoring what is bad. This would be a failure to acknowledge what is truly bad, and, in the absence of any sort of cognitive assent to the bad aspects of reality, such a disposition would be epistemically problematic.^28^ It would additionally be morally deficient because it would disable any sort of adequate engagement with what is wrong in order to attempt to correct it.

Generally, however, it seems that the most important considerations for evaluating perspectival optimism as something good or bad are its practical and moral consequences, not its epistemic rationality.^29^ Moderate forms of perspectival optimism may in fact have both practical benefit and may enrich our capacity to live morally good lives. Our intention here is not to provide a full account as to how perspectival optimism may or may not relate to practical and moral concerns, but to merely indicate that there are at least some important connections and to argue that, in some cases, perspectival optimism may be worth cultivating. Practically speaking, perspectival optimism seems to provide a focus that may help enable continued activity in the face of difficulty.

Perspectival optimism may sometimes be an enabling condition for agentive optimism and for hope.^30^ One might imagine a patient diagnosed with a difficult and troubling condition, but, because he focuses on the possible good outcome of the situation, he thus hopes for this good outcome and therefore also puts in the work necessary to undergo treatment, to take his required medicine, to undergo physical therapy, etc. Alternatively, an entrepreneur who focuses on the good aspects of situations as her business begins to develop may be more inclined to persevere and not give up. On the other hand, however, perspectival optimism, in some instances at least, might have the possibility of leading to a worse outcome. In the case of a startup business, perspectival optimism might incentivize a person to stick with a bad business idea for too long. The practical effects of perspectival optimism may often be positive but are not universally so.^31^

Let us now consider perspectival optimism’s relationship to one’s moral outlook and how it might be valuable. An illuminating example of an intentionally cultivated form of perspectival optimism is found in a story told by Iris Murdoch. Murdoch describes a mother-in-law, whom she calls (M), who uses specific acts of “attention” focusing to become more perspectivally optimistic and loving towards her daughter-in-law (D), whom she dislikes.

Although M finds D to be “unpolished,” “lacking in dignity and refinement,” “pert,” “insufficiently ceremonious, brusque, sometimes positively rude, always tiresomely juvenile,” she nevertheless tries to become more loving toward D by attempting to see D in a new light (Murdoch, 1970, p. 17). In Murdoch’s words:

M tells herself: ‘I am old-fashioned and conventional. I may be prejudiced and narrow-minded. I may be snobbish. I am certainly jealous. Let me look again.’ Here I assume that M observes D or at least reflects deliberately about D, until gradually her vision of D alters. If we take D to be now absent or dead this can make it clear that the change is not in D’s behavior but in M’s mind. D is discovered to be not vulgar but refreshingly simple, not undignified but spontaneous, not noisy but gay, not tiresomely juvenile but delightfully youthful, and so on (Murdoch, 1970, pp. 17–18)

M understands that her hostility to D is, in part, a kind of perspectival pessimism toward her. In order to be more loving, M chooses to change her perspective and make salient the good aspects of her character, which, notably, have not changed. Although M cannot simply will or force herself to love her daughter-in-law, she can attempt to change the way she sees her – in a more positive light.

Murdoch’s example shows how perspectival optimism can be an important part of a moral outlook. It can alter a person’s attitude and behavior towards others. It empowers the capacity to love the other. And on these grounds, such perspectival optimism may be worth cultivating.

Perspectival optimism is not the only form of optimism that may be worth cultivating on account of its practical benefits.^32^ Empirical research suggests that optimism helps one in attaining various goods.^33^ If the grounds for such optimism are rooted in one’s own efforts and commitment to working hard, in a way that one’s optimism is epistemically justified, then it seems valuable for one to intentionally cultivate agentive optimism as well. If one expects the future to be good because of one’s own efforts, this will likely go on to provide additional motivation to act and exert effort. With that additional effort, the action itself is more likely to attain its goal, further reinforcing one’s agentive optimism. There may well be a virtuous cycle that can come into play with agentive optimism, and the cultivation of such agentive optimism may be viewed simply as the developing a positive sense of one’s own agency. Similar dynamics may in fact be at play with generic optimism^34^ and possibly even groundless optimism. But at least in the case of agentive optimism, the positive effects of such optimism on attaining the desired ends are not necessarily accompanied by epistemic concerns, when such agentive optimism is in fact rational. Once again, agentive optimism too, viewed as a disposition, may be worth cultivating.

## Distinctions with Hope

Our discussion above of agentive optimism and perspectival optimism arguably leaves open the question as to whether and how these forms of optimism are distinct from hope, which, as noted above, has received considerably more attention in the philosophical literature than has optimism. It would be beyond the scope of this paper to review all the various conceptualizations and definitions of hope in that literature. However, we can at least offer some comparison and contrast with a few accounts of hope that perhaps most closely relate to our notions of perspectival and agentive optimism above. Thus, for example, in the account of hope given by Milona and Stockdale (2018), hope entails a desire for something good in the future and a belief that this is possible, but hope extends beyond belief and desire to include a reason for action to try to obtain the future good. Likewise, Martin (2013, p. *11)* conceives of hope not just as belief and desire, but as “a distinctive practical attitude by which we incorporate our desires for uncertain outcomes into our agency, in a specific way… standing ready to offer a certain kind of justificatory rationale for engaging in certain kinds of thought, feeling, and planning.” Again, somewhat similarly, the conception of the passion of hope offered by Thomas Aquinas is that of a “a movement of the appetitive power ensuing from the apprehension of a future good, difficult but possible to obtain; namely, a stretching forth of the appetite to such a good.”^35^ Four aspects of this understanding of hope often draw attention: that it concerns (i) a good, that (ii) is future, and (iii) difficult, but (iv) possible to obtain. The recognition of the difficulty of attaining the good may itself give rise to motivation for action.

In these accounts, hope is conceived of principally a desire, though one arising from, for example, from “the apprehension of a future good, difficult but possible to obtain.” The apprehension or belief gives rise to the desire. In contrast, optimism is not a desire, but a cognitive state concerning expectation or perspective.^36^ Like optimism, hope concerns a future possible good. Unlike expectancy-based optimism, however, hope does not necessarily entail expectation. It may, but it may not; one may hope for something that one thinks is unlikely to occur. Hope concerns some good that characteristically involves some difficulty in its being attained. In contrast, this difficulty is not necessarily characteristic of optimism.^37^

With agentive and perspectival forms of optimism, the conceptual relations are somewhat closer, but again important distinctions remain. As indicated above, perspectival optimism involves the giving of attentional focus to the positive or good aspects of an intentional object. Unlike hope, perspectival optimism can be, but need not, be future-oriented, and moreover can be, but need not be, concerned with something that is difficult. However, when *future-oriented* perspectival optimism concerns some *difficult* good, then it will in general also entail some form of hope since the very focus on such a good will also in general give rise to desire. However, what we have called perspectival optimism covers a much wider set of cases, since perspectival optimism need not involve difficulty nor, necessarily, the future, and, once again, such perspectival optimism is constituted by a cognitive state, not a desire.

There are likewise relations, but also important distinctions, between agentive optimism and hope. With agentive optimism, one forms positive expectations about a future event because at least some of that good future depends on one’s actions, and one plans to exert considerable effort towards bringing about the positive outcome. When such agentive optimism concerns some *difficult* good, then it too often entails some form of hope, as desire for that good will often be present. However, again, distinctions remain as one may hope, and plan to exert effort, even if one does not necessarily expect the good. Moreover, hope itself need not always involve one’s own personal agency. There are more passive or receptive forms of hope. One may hope for something because of the action of others. Thus, while agentive optimism concerning some difficult good will often include some form of hope, agentive optimism more broadly need not regard difficulty, and hope need not necessarily involve agency or expectation.

## Conclusion

In this paper we have proposed a new taxonomy for optimism that includes two genera and four different species or forms of optimism. The two broad genera are expectancy optimism and perspectival optimism. Resourced optimism, agentive optimism, and groundless optimism are types of expectancy optimism that are fundamentally positive expectations about the future but are differentiated by the grounds of that expectation. The genus perspectival optimism has only one type, which has the same name. We argued for the conceptual differences between these types of optimism.

We also considered the question of whether optimism can be rational. We argued that some forms of expectancy optimism – namely, resourced and agentive optimism – can sometimes be rational, and we offered two conditions or tests that must be met for any instance of optimism to be epistemically justified: the truth test and the proportionality test. These tests apply both to specific narrow optimistic beliefs, and also to wide-scoped optimism, which will often be more difficult to rationally justify. Justification of wide-scoped optimism requires a clear understanding of the scope and extent of the expectations, and of the reasons for holding these expectations. Perspectival optimism is not subject to the same sort of epistemic scrutiny as expectancy optimism, and although it is not necessarily grounded in reasons, neither is it necessarily irrational, since it is more a matter of attentional focus.

The discussion here opens up new areas of inquiry for both philosophical and empirical work. On the empirical front, it would be of interest to examine the extent to which each type of optimism considered – groundless, resourced, agentive, and perspectival – does or does not contribute to a sense of generalized optimism as captured, for example, in the Life Orientation Test Revised (LOT-R). It would also be of interest to examine whether these different forms of optimism are subsequently and differentially associated with health and other outcomes. Although the optimism literature is vast, most of the research conducted to date focuses on unrealistic optimism (whether called optimistic bias, positive illusion, the better than average effect, or other names) and generalized optimism, and less attention has been given to considering optimism that is held rationally, such as forms of resourced and agentive optimism. Likewise,additional empirical study of perspectival optimism would beof benefit to the field. We believe this taxonomy may provide researchers with a more nuanced way of understanding optimism.

Finally, by differentiating the forms of optimism and seeking to understand whether a person is holding their optimistic beliefs rationally, researchers may gain further insights into the asymmetry that exists in how people update their beliefs in response to information that is better than expected, versus information that is worse than expected (Bortolotti, 2009; Kuzmanovic et al., 2015; Sharot, 2011b). The current mechanistic account of why unrealistic optimism persists in the face of challenging information might be challenged if optimism were to be measured in more nuanced ways (See, e.g., Sharot, 2011b; Sharot et al., 2011). Of course, all of these empirical pursuits would require the development of measurement approaches to assess these different forms of optimism.

On the conceptual side, we believe the most promising area of future philosophical inquiry may be to understand more fully agentive and perspectival optimism’s practical and moral considerations, their role in attaining the good, and the extent to which their cultivation ought to be pursued, along with when such forms of optimism may be problematic. Philosophy has often derided or ignored the phenomenon of optimism, and for groundless expressions of optimism this was done with good reason. This paper, however, has shown that rational forms of optimism can be manifest and are worth our consideration. These forms of optimism could play an important role in human flourishing and deserve greater philosophical attention.

## Data Availability

Not applicable.

## Footnotes

1 See, amongst others, Day (1969), Pettit (2004), Martin (2013), Kadlac (2015), Eagleton (2015), Callina et al. (2018).

2 For example, Scruton (2010).

3 Psychologists have studied the concept of optimistic biases or “unrealistic optimism” for now over 40 years. Shepperd et al. (2013) identified 984 at the publication of their paper, and there have been many more publications since.

4 This literature demonstrating the positive benefits of optimism is vast. For seminal works in this regard, see Taylor (1989), and Taylor and Brown (1988, 1994). More recent works summarizing these benefits include Alicke and Sedikides (2009) Carver et al. (2010); Sharot, 2011a, b; Bortolotti and Antrobus, (2015), *and* Avvenuti et al. (2016). For two recent meta analyses studying optimism and all-cause mortality see: Craig et al. (2021); Rozanski et al. (2019). Some biologists maintain that optimism may have evolutionary adaptive benefits (See McKay & Dennett, 2009; Varki, 2009).

5 Eagleton (2015). This sentiment is congruent with a long line of historical thinkers including Voltaire, Nietzsche, and Huxley, and more modern thinkers who have engaged in the “ethics of belief” debates (See e.g., .

6 The distinction between epistemic and instrumental rationality is not uncontroversial. For example, Plantinga distinguishes five ‘varieties’ of rationality (1993) and Goldman (1986) explicitly questions the usefulness of the distinction. Some epistemologists and philosophers of science have attempted to reduce epistemic rationality to instrumental rationality (See e.g., Foley (1987, 1993), Laudan (1990) Nozick (1993) and Kitcher (1992). Debating this distinction is far beyond the scope of this paper, and we are persuaded by Kelley’s (2003) critique of those who try to collapse the distinction between epistemic and instrumental rationality, so we assume the distinction holds and our interest is primarily in the “rationality” of optimism on its epistemic, not it’s instrumental, merits.

7 The distinction between epistemic rationality and instrumental rationality is not often discussed in the empirical literature. Bortolotti (2018) and Jefferson et al. (2017) specifically focus on the epistemic nature of the deficiency in holding unwarranted optimistic beliefs. Others, such as Taylor (1989) seem to collapse epistemic rationality into instrumental rationality when then accept so-called positive illusions as normal and only calling those “excessive” that typically lead to bad outcomes. For a comprehensive examination of the epistemic status of delusions, see Bortolotti (2009).

8 Bury et al. also consider hope and optimism to be a matter of degree, in terms of probability.

9 Psychologists distinguish dispositional or “trait” optimism from “attributional” or “explanatory” style optimism. Because explanatory style optimism describes how people make judgements about the causes of prior events, we are not convinced that “optimism” is a felicitous description of this phenomenon. We do not discuss it at length here, but we do identify this as an area for future research in our conclusions. We omit it here because it would take a lengthy philosophical argument to show why this phenomenon has been improperly termed “optimism.” For examples of this literature, see: Gillham et al. (2001); Peterson and Steen (2002).

10 Unrealistic optimism is not uncontroversial in the psychological literature (e.g., Harris & Hahn, 2011; see also e.g., Chambers et al., 2003; Moore & Small, 2008).

11 Prior to Shepperd, most “unrealistic optimism” regarded expectations that a positive outcome for oneself as compared to “average” or the population and was referred to as “comparative optimism” in (Chambers et al., 2003). This is what Shepperd et al. (2013) and Jefferson et al. (2017) refer to as “comparative” unrealistic optimism, which we explain in what follows.

12 This same distinction is found in Jefferson et al. (2017).

13 As another example, students frequently overestimate their likelihood of living until 80 years old (Harris & Hahn, 2011).

14 A similar and related phenomenon is known as the “better than average effect”, which is “the proclivity to rate one’s current abilities, attributes, or personality traits more favorably than those of the average peer.” (Zell et al., 2020, p. 119). See also, Brown (2012).

15 This has been noted by many. E.g., see Weinstein (1980), Shepperd et al. (2013), and Jefferson et al. (2017).

16 For many more examples, see Shepperd et al. (2013). This statistic cited could be viewed as erroneous depending on how one interprets “average.” We simply note that it is impossible for 70% to be less likely to divorce than the average (median) person. For an interesting discussion of statistical issues related to comparative optimism, see (Harris & Hahn, 2011).

17 It is perhaps open to dispute whether, in the end, comparative unrealistic optimism in fact entails judgements of objective likelihoods, which would then make this a distinction without a difference. Moore & Small (2008) make a specific prediction that (rational) absolute pessimism will often co-occur with comparative optimism.

18 See Jefferson et al. (2017, p. 4). Borolotti (2018, p. 523) makes a similar point.

19 Bortolotti (2018, p. 523) similarly suggests a departure from the language of “unrealistic,” but she prefers to call unrealistic optimism “optimistically biased beliefs.”

20 Although grounded, this does not mean that such a person’s optimism is justified. It could still be unjustified or “unrealistic” due to overconfidence (Benoît & Dubra, 2011; Moore & Healy, 2008), egocentrism (Shepperd et al., 2013; Weinstein, 1980), or other factors. We discuss the rationality of resourced and agentive optimism in the next section.

21 Even those who deny that optimism is a belief state and claim that it is more like a “can do attitude,” or a hope (see Flanagan, 2009), still make expectancy about the future core to their concept.

22 https://dictionary.cambridge.org/us/dictionary/english/optimism.

23 https://www.merriam-webster.com/dictionary/optimism.

24 A lack of proportionality could be explained using a variety of additional cognitive or psychological descriptions, such as “a distortion in personal risk assessment, a distortion in the perceived risk of the target,…motivational sources such as a desire to deny vulnerability to harm,…egocentric thinking” (see Shepperd et. all, 2003, p. 396). See other known biases in judgement in Moore and Small (2007).

25 There can also be, in principle, cases in which the optimistic belief is itself a “game-changer” such that the belief contributes to the realization of the positive outcome so that, counterfactually, with the optimistic belief in place the positive outcome is enabled, or rendered more likely. If the optimistic belief were not in place, then the positive outcome would not come about. One might imagine a setting in which a belief that one’s public speech will turn out well in fact effectively contributes to its doing so. See Bortolotti (2018), Snow (2018*), and* Taylor and Sherman (2008) for related discussion concerning situations in which even misbeliefs might sometimes be adaptive in this way. Relatedly, see Marušić (2015) for cases in which it might in some sense be advantageous for people to believe they will do various things even when they have access to evidence that they will not.

26 As noted previously, there may be instrumental or prudential reasons to hold wide-scoped optimistic beliefs.

27 Kierkegaard is an example of a religious thinker who thought that certain positive “expectancies”, or expectations, were entailed by having faith in God. Thus, he has an essay titled, “The Expectancy of Faith.” For an analysis of how expectancy is an integral component of Kierkegaardian faith, see Wilson & Evans (2019, pp. 377-381).

28 As noted in footnote 9, psychologists have identified a so-called “explanatory style optimism” to be a kind of optimism that concerns whether a person sees their successes as likely to proceed into the future (stable), due to themselves (internal), and pertaining to all aspects of life (global), and their failures as temporary, due to external causes (external), and only specific to the present situation (local). One might consider the internal/external dimension of this explanatory style optimism a form of perspectival optimism that specifically concerns focusing on the positive aspects of one’s agency within one’s own successes and failures. However, we do not think this is a paradigm case of perspectival optimism, and it may even be morally vicious (something better characterized as a lack of proper responsibility or accountability, or worse, narcissism). See footnote 9 for further references on explanatory style optimism.

29 For an overview of the concept and issues surrounding practical rationality, as well as an extended bibliography, see Wallace (2020).

30 For more specifics on the relationship between hope and optimism, see the next section.

31 The psychology literature is filled with examples of how unrealistic optimism can be both practically beneficial but also have negative consequences in some areas of life. See, e.g., *Sharot (2011)*. It is an open question as to whether a disposition to perspectival optimism alone (i.e. not being accompanied by a disposition to general optimism or unrealistic optimism) would lead to fewer negative consequences because there is no accompanying epistemic deficiency. If a measure were developed to distinguish these forms of optimism, this could be a positive development for the field.

32 Researchers have found that dispositional optimism can be boosted, at least temporarily, through mental exercises and habit formation. See, Carver and Scheier (2014).

33 For the most recent reviews of optimism’s positive benefits, including engaging in healthy behaviors, abstaining from smoking and drug use, problem focused coping, planning, seeking social support, and reduced all-cause mortality, see Carver et al. (2010), Craig et al. (2021, p. 2), Rozanski et al. (2019), and Sharot (2011b).

34 Carver & Scheier (1998) relate such positive expectations about the future to goals and argue that a greater willingness to act is a consequence of optimism. Similar arguments have been made by Bortolotti (2018), Taylor and Sherman (2008) and virtuous cycles are noted in Carver et al. (2010)

35 Summa Theologiae I.II.40.2.

36 There is, however, arguably more conceptual overlap when considering hope and optimism as traits or dispositions. As dispositions, we might define hope, drawing upon the accounts above, as “a disposition to fix one’s attention on the possibility of some future desired good, characteristically in the face of difficulty” and expectancy-based optimism as “a disposition towards having expectations that the future will be good.” Even understood as dispositions, there are again still important distinctions in terms of hope. Once again, hope, but not necessarily optimism, characteristically involves goods that are difficult to obtain; and optimism, but not necessarily hope, entails an expectation that the good will come about.

37 This is so on conceptual grounds, and there is also empirical evidence supporting this as well (Bury et al., 2016).
